# Anti-Biofilm and Immunomodulatory Activities of Peptides That Inhibit Biofilms Formed by Pathogens Isolated from Cystic Fibrosis Patients

**DOI:** 10.3390/antibiotics3040509

**Published:** 2014-10-20

**Authors:** César de la Fuente-Núñez, Sarah C. Mansour, Zhejun Wang, Lucy Jiang, Elena B.M. Breidenstein, Melissa Elliott, Fany Reffuveille, David P. Speert, Shauna L. Reckseidler-Zenteno, Ya Shen, Markus Haapasalo, Robert E.W. Hancock

**Affiliations:** 1 Centre for Microbial Diseases and Immunity Research, Department of Microbiology and Immunology, University of British Columbia, Vancouver, BC, V6T 1Z4, Canada; E-Mails: cesar@hancocklab.com (C.D.L.F.-N.); sarah@hancocklab.com (S.C.M.); l.jiang89@gmail.com (L.J.); elena.breidenstein@pasteur.fr (E.B.M.B.); melissaelliott0@gmail.com (M.E.); fany.reffuveille@gmail.com (F.R.); 2 Division of Endodontics, Department of Oral Biological and Medical Sciences, Faculty of Dentistry, University of British Columbia, Vancouver, BC, V6T 1Z3, Canada; E-Mails: zhejun@dentistry.ubc.ca (Z.W.); yashen@dentistry.ubc.ca (Y.S.); markush@dentistry.ubc.ca (M.H.); 3 Department of Pediatrics, University of British Columbia, Vancouver, BC, V6H 3V4, Canada; E-Mail: dspeert@cfri.ubc.ca; 4 Athabasca University, Athabasca, AB, T9S 3A3, Canada; E-Mail: shaunaz@athabascau.ca

**Keywords:** anti-biofilm, immunomodulation, peptides, antibiotic-resistance, cystic fibrosis

## Abstract

Cystic fibrosis (CF) patients often acquire chronic respiratory tract infections due to *Pseudomonas aeruginosa* and *Burkholderia cepacia* complex (Bcc) species. In the CF lung, these bacteria grow as multicellular aggregates termed biofilms. Biofilms demonstrate increased (adaptive) resistance to conventional antibiotics, and there are currently no available biofilm-specific therapies. Using plastic adherent, hydroxyapatite and flow cell biofilm models coupled with confocal and scanning electron microscopy, it was demonstrated that an anti-biofilm peptide 1018 prevented biofilm formation, eradicated mature biofilms and killed biofilms formed by a wide range of *P. aeruginosa* and *B. cenocepacia* clinical isolates. New peptide derivatives were designed that, compared to their parent peptide 1018, showed similar or decreased anti-biofilm activity against *P. aeruginosa* biofilms, but increased activity against biofilms formed by the Gram-positive bacterium methicillin resistant *Staphylococcus aureus*. In addition, some of these new peptide derivatives retained the immunomodulatory activity of 1018 since they induced the production of the chemokine monocyte chemotactic protein-1 (MCP-1) and suppressed lipopolysaccharide-mediated tumor necrosis factor-α (TNF-α) production by human peripheral blood mononuclear cells (PBMC) and were non-toxic towards these cells. Peptide 1018 and its derivatives provide promising leads for the treatment of chronic biofilm infections and hyperinflammatory lung disease in CF patients.

## 1. Introduction

Bacteria form biofilms when growing on surfaces or air-liquid interfaces. Biofilms are structured aggregates of bacteria encased in a protective extracellular matrix that can contain polysaccharides, proteins, extracellular DNA, and lipids [1]. The switch from a free swimming, planktonic to an adherent biofilm lifestyle results in increased adaptive resistance to antimicrobial agents making biofilm-related infections inherently difficult to treat [1,2]. Apart from colonizing inert materials such as catheters and medical implants, bacterial biofilms are prevalent in chronic infections, such as those that develop in the lungs of cystic fibrosis (CF) patients [3,4]. CF is the most common eventually fatal autosomal-recessive disorder in Caucasian populations and is caused by mutations in the CF transmembrane conductance regulator (CFTR) chloride-channel, as well as various modifier genes that influence severity. CF patients rapidly acquire lifelong chronic respiratory infections that lead to hyper-inflammation and progressive destruction of lung function [5]. 

The opportunistic pathogen *P. aeruginosa* is the most prevalent pathogen associated with lung deterioration in patients with CF [3,5]. Its high intrinsic resistance and ability to develop biofilms in the CF lung, combined with the lack of specific anti-biofilm therapeutics, seriously hinders the treatment of chronically-infected CF patients, and although *P. aeruginosa* can initially be transiently suppressed by aggressive antibiotic therapy, the same strain can re-emerge and predominate for years. In fact, it has been suggested that treatment with antibiotics such as tobramycin (used in the treatment of CF), tetracycline, and norfloxacin might contribute to infection chronicity by inducing biofilm formation [6,7]. During its long-term colonization of the CF lung, *P. aeruginosa* undergoes several genetic adaptations leading to different colony morphologies [8], some of which are linked to bacterial persistence. For example, mucoid *P. aeruginosa* strains*,* which result from mutations within the anti-sigma factor *mucA*, have been associated with biofilm formation [9] and antibiotic resistance [10]. Likewise, small colony variant (SCV) colonies have been shown to be hyper-adherent with reduced susceptibility to antibiotics [11,12,13]. Another major factor is the emergence of mutator strains that enable the rapid evolution of resistant strains in the CF lung. The biofilm mode is also associated with resistance to phagocytic killing, which is one of the major mechanisms of clearance in the lung. In addition to *P. aeruginosa*, infection with bacterial species from the *Burkholderia cepacia* complex (Bcc), a collection of nine genotypically distinct but phenotypically similar species, has been associated with a poor prognosis in CF patients [14,15].

Cationic host defense (antimicrobial) peptides represent a novel alternative to conventional antibiotics. These peptides, e.g., the human cathelicidin LL-37, often have modest direct antimicrobial activities for planktonic cells [16] and are known to possess immunomodulatory properties [17]. More recently, it has been demonstrated that LL-37 has excellent anti-biofilm activity *vs.* *P. aeruginosa* causing a 50% decrease in biofilm formation at one sixteenth the minimum inhibitory concentration (MIC) [18]. Similar results were subsequently shown by other groups [19,20]. This encouraged the development of improved synthetic cationic peptides with activity against bacterial biofilms [21,22,23,24,25,26,27].

Anti-biofilm peptides are biochemically similar to antimicrobial peptides in that they are short (12–50 amino acids long) and amphipathic, having two to nine basic residues (R or K) and ~50% hydrophobic residues [18,19,20,21,22]; however they exhibit specific activity against biofilms at concentrations often well below their MIC for planktonic cells [18,19,20,21,22], and there is no correlation between anti-biofilm activity and anti-planktonic cell activity [18]. Previously identified anti-biofilm peptides include peptide 1037 that was shown to inhibit biofilm formation by both Gram-negative and Gram-positive bacteria [21]. More recently, peptide 1018 (VRLIVAVRIWRR-NH_2_; also called IDR-1018), developed initially as an immune modulator [28,29,30], was demonstrated to have potent broad-spectrum anti-biofilm activity that mechanistically involved the binding and degradation of (p)ppGpp nucleotides, which are involved in biofilm formation and maintenance [22]. Peptide 1018 has also been shown to synergize with conventional antibiotics to treat biofilms formed by multiple bacterial species [27]. These properties indicate that 1018 might be a useful lead for the treatment of hyperinflammatory chronic biofilm infections in the context of CF.

Here we tested the anti-biofilm activity of peptide 1018 against a variety of *P. aeruginosa* and *Burkholderia* strains isolated from CF patients. Further, we designed a variety of 1018 derivatives and assessed their anti-biofilm and immunomodulatory properties. Since persistent lower airway infection and inflammation is a significant cause of mortality and morbidity in cystic fibrosis [31], the combined immunomodulatory and anti-biofilm effects of the given peptides provide promising leads for decreasing infections and improving lung pathology and the overall course of the disease.

## 2. Results

### 2.1. MICs vs. Cystic Fibrosis Isolates

Peptide 1018 has previously been shown to have activity both as an immune modulator [28,29,30] and as an anti-biofilm agent [22,27] against individual strains from several species of bacteria including *P. aeruginosa* and *Burkholderia cenocepacia*. The latter observation was particularly surprising since planktonic *B. cepacia* complex strains are known to be completely resistant to the effects of cationic agents like polymyxin B and cationic antimicrobial peptides by virtue of their lack of a self-promoted uptake system across the outer membrane [32]. Here we extended our observations on the anti-biofilm activity* vs.* *Burkholderia* using a broad variety of *B. cepacia* complex species from CF patients and also examined activity* vs.* a broad range of *P. aeruginosa* chronic pulmonary infection CF isolates, since these are known to form biofilms in the CF lung. MIC assays were performed to determine the direct antimicrobial activity of peptide 1018 against planktonic cells. Peptide 1018 exhibited somewhat better planktonic antimicrobial activity* vs.* *Pseudomonas* compared to human host defense peptide LL-37 [18] and synthetic cationic peptide 1037 [21] (Table 1), but was still quite weak with MICs ranging from 16 to 32 μg/mL. Interestingly, all *P. aeruginosa* clinical isolates showed higher MICs for human cathelicidin LL-37 compared to reference strains PAO1 and PA14, confirming the idea that this peptide has little useful activity* vs.* planktonic strains (Table 1). In contrast cationic antibiotic polymyxin B had MICs of 2–4 μg/mL indicating that the low activity did not *per se* reflect resistance to cationic peptides.

**Table 1:** Peptide 1018 demonstrated weak activity* vs.* planktonically-grown *P. aeruginosa* cystic fibrosis strains and no activity* vs.* *B. cepacia* complex cystic fibrosis isolates. All minimum inhibitory concentration (MIC) assays were performed at least three times. For comparison we included the previously studied *P. aeruginosa* (non-cystic fibrosis) wild type strains PAO1 and PA14 and *B. cenocepacia* 4813 [22], as well as showing results for two previously described anti-biofilm peptides (LL-37 [18] and 1037 [21]) and the cationic lipopeptide polymyxin B. Different *Burkholderia* genomovars denote strains which are phylogenetically differentiable but phenotypically indistinguishable. *Burkholderia multivorans* (genomovar II), genomovar III (divided into two *recA* clusters, III-a and III-b, *Burkholderia stabilis* (genomovar IV), *Burkholderia dolosa* (genomovar VI), *Burkholderia ambifaria* (genomovar VII).

Strains (Colonial Morphology or Genomovar)	MIC (μg/mL)			
	1018	LL-37	1037	Polymyxin B
*P. aeruginosa* PA01	16	32	304	2
*P. aeruginosa* PA14	16	32	304	2
*P. aeruginosa* 3195 (Classic)	16	64	304	2
*P. aeruginosa* C2773 (Classic)	16	256	152	4
*P. aeruginosa* 1172 (Classic)	16	>128	304	2
*P. aeruginosa* 7632 (Classic)	16	256	304	2
*P. aeruginosa* 3330 (Classic)	16	>128	304	2
*P. aeruginosa* 4020 (Classic)	16	>128	304	2
*P. aeruginosa* C4276 (Classic)	32	256	608	2
*P. aeruginosa* 2631 (Classic)	32	>256	608	4
*P. aeruginosa* C4278 (Mucoid)	16	>256	304	2
*P. aeruginosa* 4608 (Mucoid)	32	>128	304	2
*P. aeruginosa* 2639 (Mucoid)	32	>128	608	2
*P. aeruginosa* 7633 (Entire)	16	>128	304	2
*P. aeruginosa* 2955 (Dwarf)	50	-^a^	-	-
*B. multivorans* D2661 (Genomovar II)	>512	>512	>608	>128
*B. cenocepacia* 4813 (IIIa)	>256	>256	>608	>128
*B. cenocepacia* C5424 (IIIa)	128	>256	>608	>128
*B. cenocepacia* CEP0055 (IIIb)	128	>512	>608	>128
*B. cenocepacia* CEP509 (IIIb)	>256	>512	>608	>128
*B. stabilis* C6061 (IV)	64	>512	304	>128
*B. dolosa* CEP0021 (VI)	>256	>512	>608	>128
*B. ambifaria* CEP0996 (VII)	64	>512	>608	>128

^a^ Means not done.

Consistent with previous studies [22,32], *Burkholderia* was confirmed to be intrinsically resistant to antimicrobial peptides and polymyxin B by the MIC assays (Table 1). Only peptide 1018 was able to affect any *Burkholderia* planktonic cells, demonstrating relatively high MICs of 64–128 µg/mL* vs.* five out of the total of 11 known genomovars. Even peptides known to exhibit very potent direct planktonic antimicrobial activity (HHC-10 and HHC-36) [33] were found to be inactive against planktonic cells of *Burkholderia* strains (MIC > 256 µg/mL).

### 2.2. Anti-Biofilm Activity vs. Cystic Fibrosis Isolates

To assess the biofilm-forming ability of the different clinical isolates, dilutions (1/100) of overnight cultures were incubated in 96-well polypropylene plates using BM2 glucose medium, as previously described [22]. No apparent correlation was observed between the different bacterial morphologies and the formation of biofilm biomass of *P. aeruginosa* isolates (Table 2). Three *P. aeruginosa* strains with classical morphology C2773, 3195, and 1172, and mucoid strain 2639 exhibited increased biofilm formation when compared to reference strains PA01 and PA14 (Table 2). In contrast, strains *B. cenocepacia* CEP0055 and *B. ambifaria* CEP0996 produced about a third of the biofilm compared to the previously-studied strain *B. cenocepacia* 4813 (Table 2). Overall, five of the eight tested *Burkholderia* strains showed relatively weak biofilm formation on the surface of the microtiter wells (Table 2).

**Table 2:** Activity of peptide 1018 against biofilms formed by *P. aeruginosa* (PA) and *Burkholderia* (B) clinical isolates from cystic fibrosis (CF) patients. Usually three, but at least two independent experiments and eighteen replicates were performed per condition. The differentiating colony morphology or genomovar is given in brackets in the first column. Strains PAO1 and PA14 are reference strains that have been fully sequenced.

Strains (Colonial Morphology or Genomovar)	Biofilm Formation ^a^	Inhibition by 10 µg/mL 1018
*P. aeruginosa* PA01	0.79 ± 0.22	51%
*P. aeruginosa* PA14	0.82 ± 0.21	49%
*P. aeruginosa* 3195 (Classic)	0.99 ± 0.16	37%
*P. aeruginosa* C2773 (Classic)	0.89 ± 0.17	60%
*P. aeruginosa* 1172 (Classic)	0.92 ± 0.27	37%
*P. aeruginosa* 7632 (Classic)	0.59 ± 0.12	19%
*P. aeruginosa* 3330 (Classic)	0.84 ± 0.20	33%
*P. aeruginosa* 4020 (Classic)	0.62 ± 0.16	53%
*P. aeruginosa* C4276 (Classic)	0.62 ± 0.28	53%
*P. aeruginosa* 2631 (Classic)	0.74 ± 0.27	44%
*P. aeruginosa* C4278 (Mucoid)	0.73 ± 0.20	58%
*P. aeruginosa* 4608 (Mucoid)	0.62 ± 0.09	67%
*P. aeruginosa* 2639 (Mucoid)	0.93 ± 0.20	44%
*P. aeruginosa* 7633 (Entire)	0.72 ± 0.16	49%
*P. aeruginosa* 2955 (Dwarf)	0.64 ± 0.19	41%
*B. multivorans* D2661 (Genomovar II)	0.75 ± 0.07	75%
*B. cenocepacia* 4813 (IIIa)	0.94 ± 0.18	52%
*B. cenocepacia* C5424 (IIIa)	0.75 ± 0.18	59%
*B. cenocepacia* CEP0055 (IIIb)	0.33 ± 0.11	58%
*B. cenocepacia* CEP509 (IIIb)	0.42 ± 0.08	61%
*B. stabilis* C6061 (IV)	0.39 ± 0.07	53%
*B. dolosa* CEP0021 (VI)	0.41 ± 0.06	55%
*B. ambifaria* CEP0996 (VII)	0.29 ± 0.10	62%

^a^ Values shown correspond to mean ± SD absorbance_595_ readings after crystal violet staining of biofilms adherent to the wells of microtiter growth trays.

These strains were then challenged with sub-inhibitory levels of anti-biofilm peptide 1018. At 10 µg/mL, the peptide was able to reduce PA01 and PA14 biofilm formation by ~50% (*p* < 0.05; Table 2). Similarly, biofilms formed by different *P. aeruginosa* clinical isolates were also susceptible to the peptide (Table 2). Biofilms formed by classic morphology strains C2773, C4276, and 4020, and mucoid strains C4278 and 4608 were inhibited by at least 50% by the peptide (Table 2). On the other hand, biofilms produced by with classic strains 3195, 7632, 3330, 1172, and 2631, mucoid strain 2639, and dwarf (*i.e.*, small-colony variant) strain 2955 were somewhat less susceptible to the peptide (Table 2). Biofilm biomass was also significantly reduced by 52%–75%, upon peptide challenge in *Burkholderia* strains belonging to different genomovars, and this appeared to be independent of the relative ability of each strain to form biofilms (Table 2), as well as the resistance of planktonic cells to treatment with peptide 1018 (Table 1).

### 2.3. Microscopic Imaging of Anti-Biofilm Activity

**Figure 1:**
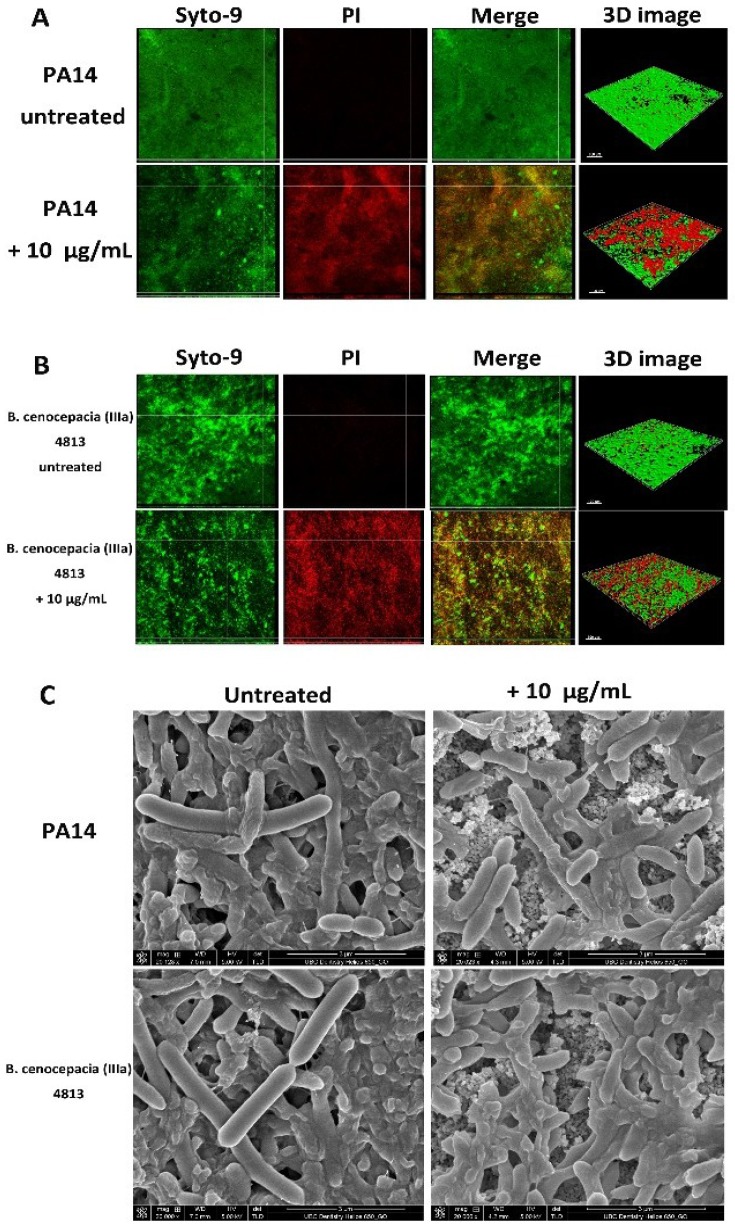
Sub-MIC concentrations of anti-biofilm peptide 1018 triggered biofilm cell death in the hydroxyapatite biofilm model. (A and B) Biofilms were stained using SYTO-9 to stain live cells green and propidium iodide (PI) to stain dead cells red. Merged images showing bacteria stained with both SYTO-9 and PI are also shown, and a yellow or red merged color was observed for dead cells. Samples were then examined using confocal laser scanning microscopy (CLSM). Each panel shows reconstructions from the top in the large panel and sides in the right and bottom panels (xy, yz, and xz dimensions). Three-dimensional reconstructions of biofilms are shown on the far right panel (3D image). The scale bar represents 100 µm in length. (**A**) *P. aeruginosa* PA14 untreated and treated with 10 µg/mL of peptide; (**B**) *B. cenocepacia* (IIIa) 4813 untreated and treated with peptide. Two independent experiments were performed for each condition; (**C**) SEM images showing the effect of peptide treatment on biofilm cells. *P. aeruginosa* and *B. cenocepacia* biofilms were developed on hydroxyapatite (HA) discs for 3 days and treated three times (every 24 h) with 10 µg/mL of peptide 1018. Biofilms were observed using SEM at a magnification of 20,000× operating at 5 kV. Untreated biofilms grown using the HA biofilm model were typically 30–40 μm thick, while biofilm thickness was reduced to 10–20 μm in peptide-treated samples.

The ability of peptide 1018 to inhibit biofilms was further investigated using hydroxyapatite (HA) discs as a biologically-relevant biofilm substratum [34]. *P. aeruginosa* PA14 (Figure 1A) and *B. cenocepacia* 4813 (Figure 1B) were resuspended in brain-heart infusion broth (BHI) and grown for 3 days on HA disks. Subsequently the biofilm coated HA disks were treated or not with 10 µg/mL of peptide. Fresh BHI medium and peptide were added every 24 h for a total of 3 days, and the effect of the peptide was evaluated using confocal microscopy and scanning electron microscopy. Samples for confocal microscopy were rinsed in 0.85% physiological saline for 2 min to remove the culture broth. After live/dead cell staining, five random areas of the biofilm on each disk were examined. Treatment with peptide 1018 significantly increased the number of dead biofilm cells to around 50% for both pathogens (Table 3, Figure 1). Interestingly, peptide-induced cell death was observed in *B. cenocepacia* biofilms grown on hydroxyapatite discs, which differed from previous results obtained using the flow cell methodology in which no major increase in cell death was recorded [20]. Additional experiments using scanning electron microscopy further supported the notion that the peptide killed biofilm cells, as cell death was apparent from the substantial amount of extracellular debris (presumably from compromised cells) accumulated within the biofilm (Figure 1C). Also, biofilm cells exhibited disrupted morphologies and were smaller in size in the treated samples (Figure 1C).

**Table 3:** Proportion of dead biofilm cells in hydroxyapatite plates after treatment with sub-MIC levels of peptide 1018. Biofilms were grown in HA discs for 3 days and treated with peptide 1018 every 24 h. After scanning confocal microscopy the volume ratio of red fluorescence (due to the dead cell stain propidium iodide to green (all bacteria stain Syto-9) plus red fluorescence indicated the proportion of killed cells. Three independent assays were performed. Different superscript letters indicate statistically significant differences between groups (*p* < 0.05).

Bacterial Strains	Proportion of Dead Cells (%)	
	Untreated	10 µg/mL 1018
*P. aeruginosa* PA14	7 ± 4	48 ± 17^ a^
*B. cenocepacia* (IIIa) 4813	6 ± 3	42 ± 13^ a^

^a^ Statistically significant differences (*p* < 0.05) were found between untreated and 1018-treated *P. aeruginosa* PA14 and *B. cenocepacia* (IIIa) 4813.

### 2.4. Derivatives of 1018: Anti-Biofilm and Immunomodulatory Activity

**Figure 2:**
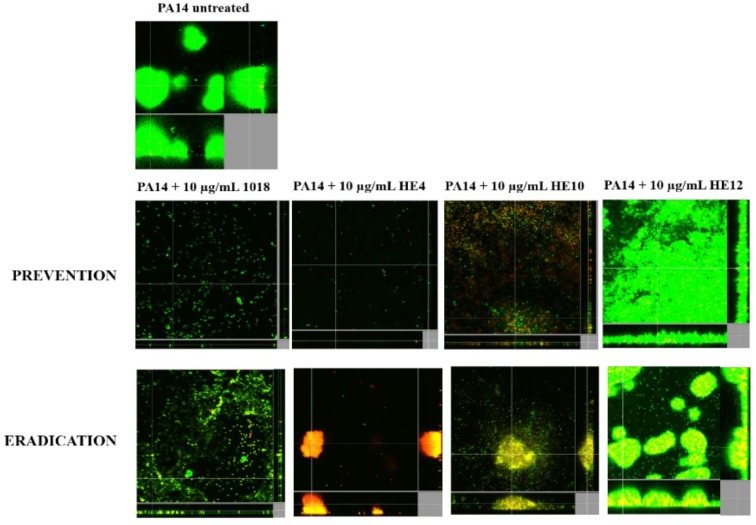
Anti-biofilm activity of peptide 1018 derivatives against *P. aeruginosa* PA14 in flow cells. For biofilm prevention conditions, the peptides were added to the medium from the beginning of the experiment. For eradication experiments, bacteria were allowed to develop structured 2-day-old biofilms prior to peptide and antibiotics treatment for the following 24 h. After 3 days, bacteria were stained green with the all bacteria stain Syto-9 and red with the dead-bacteria stain propidium iodide (merge shows as yellow to red) prior to confocal imaging. Each panel shows reconstructions from the top in the large panel and sides in the right and bottom panels (xy, yz, and xz dimensions).

As peptide 1018 has demonstrated potent anti-biofilm activity against diverse bacterial species [22] and excellent immune modulatory activity [28,29,30], we attempted to investigate structure-activity relationships by designing peptides based on the amino acid sequence of 1018 but with various amino acid substitutions (Table 4), designed to test the effects of sequence inversion and especially altering the five amino acid hydrophobic stretch L_3_IVAV_7_. Interestingly, inverting the sequence of 1018 in peptide HE1 maintained the anti-biofilm activity *vs.* *P. aeruginosa* and only slightly reduced it against *B. cenocepacia*. In contrast, altering three amino acids including switching an R and V residue in the sequence substantially reduced activity, which was also true for the reversed sequence in peptide HE2. These initial screens also identified peptides HE4 and HE10 as two of the more active anti-biofilm peptides, and a scrambled shorter peptide HE12 as a virtually inactive peptide (Figure 2) that we subsequently used as a negative control.

**Figure 3:**
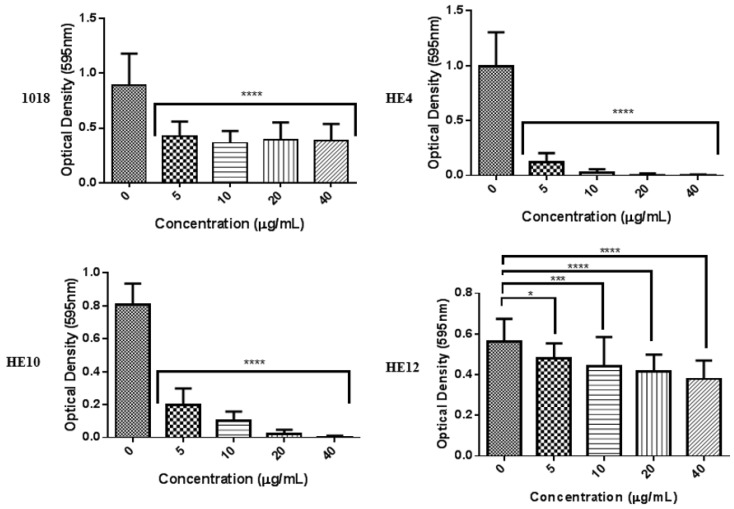
Biofilm inhibitory activity against methicillin resistant *Staphylococcus aureus* (MRSA) in 96-well plates. Dilutions (1/100) of overnight cultures were incubated in BM2 biofilm-adjusted in polypropylene microtiter plates (Fisher Scientific, Waltham, MA, USA) in the presence of the different peptides for 24 h at 37 °C. Planktonic cells were removed, biofilm cells adhering to the side of the tubes were stained with crystal violet, and absorbance at 595 nm was measured using a microtiter plate reader (Bio-Tek Instruments Inc., Winooski, VT, USA).

The three most effective 1018 derivatives were then further evaluated for anti-biofilm activity against *P. aeruginosa* PA14 using the flow cell method (Figure 2). Peptides HE4 and HE10 demonstrated strong anti-biofilm activity comparable with that of 1018, while HE12 was substantially less active against *P. aeruginosa* biofilms, both under inhibition and eradication conditions (Figure 2). We then considered whether these peptide derivatives had conserved the ability of peptide 1018 to inhibit biofilms formed by Gram-positive bacteria. Interestingly, HE4 and HE10 were notably more active against methicillin-resistant *Staphylococcus aureus* (MRSA) compared to 1018 under static conditions, while HE12 showed less activity (Figure 3). These results demonstrate that the broad-spectrum activity displayed by 1018 [22] is generally conserved in these 1018 derivatives.

**Table 4:** Anti-biofilm activity of peptides derived from parent peptide 1018. In bold are shown the different amino acid substitutions made to parent peptide 1018. Hyphens indicate amino acid residues that were deleted in relation to peptide 1018. For peptides 1018, HE4, HE10, and HE12 the concentration used was 10 µg/mL. These studies were performed with a Bioflux apparatus resulting in the higher activity* vs.* *Pseudomonas*. For peptides HE1, HE2, and HE3, 15 µg/mL of peptide was used since 10 µg/mL of peptide showed low activity. The concentration of peptide used in each case was lower than the MIC for both *P. aeruginosa* (e.g., MICs for HE-4 and HE-12 *vs*. PA14 were 80 µg/mL) and *B. cenocepacia* (MICs > 256 µg/mL).

Peptide	Sequence ^a^	Modification	Anti-Biofilm Activity (% Decrease in Biofilm)	
			*P. aeruginosa* PA14	*B. cenocepacia* 4813
1018	VRLIVAVRIWRR	Parent	99%	52%
HE1	RRWIRVAVILRV	Reverse sequence	93%	73%
HE2	VRLI**R**AVR**A**WR**V**	Break hydrophobicity; maintain charge	42%	24%
HE3	**V**RW**A**RVA**R**ILRV	Reverse of HE2	31%	21%
HE4	VRLI**W**AVRIWRR	Substitute in another W for V	99%	- ^b^
HE10	VRLI--VRIWRR	Truncate to remove hydrophobic patch	39%	-
HE12	RFKRVARVIW	Scrambled smaller peptide	10%	-

^a^ Single amino acids sequence. In bold are shown the different amino acid substitutions made to parent peptide 1018; ^b^ Means not done.

Statistical significance was assessed using one-way ANOVA (*, *p* < 0.05; ***, *p* < 0.001; ****, *p* < 0.0001).

**Figure 4:**
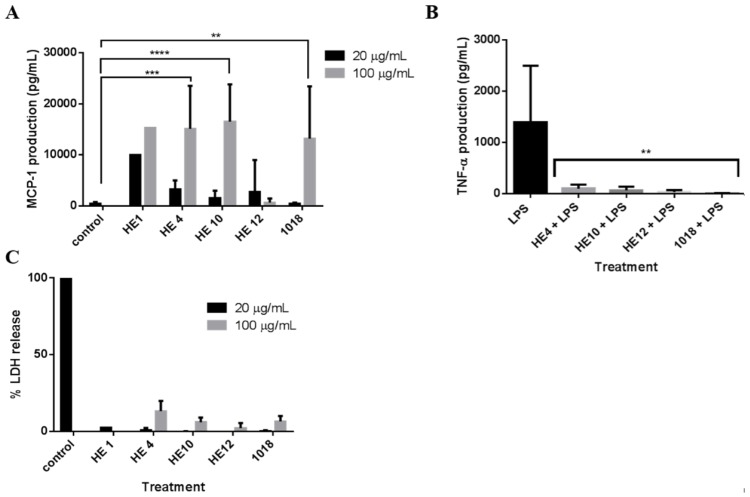
Immunomodulatory activity and cytotoxicity of peptide 1018 and its derivatives HE1, HE4, HE10, HE12. (**A**) Human peripheral blood mononuclear cells were subjected to 20 µg/mL and 100 µg/mL of HE1, HE4, HE10, HE12, and 1018 for 24 h. Monocyte chemotactic protein-1 (MCP-1) production was assessed using sandwich enzyme linked immunosorbent assay (ELISA). The level of MCP-1 induction was similar for HE1, HE4, HE10, and 1018 at 100 µg/mL; (**B**) LPS (20 ng/mL) was co-administered with 20 µg/mL of each of the aforementioned peptides for 24 h. The tumor necrosis factor-alpha (TNF-α) production in response to both stimulants was assessed using enzyme linked immunosorbent assay (ELISA) and compared to lipopolysaccharides (LPS) alone. (The peptides themselves did not induce any significant TNF-α production). In both A and B, statistical significance was assessed using one-way ANOVA (**, *p* < 0.01; ***, *p* < 0.001); (**C**) Peptide 1018 and its derivatives were not cytotoxic. Lactate dehydrogenase release from peripheral blood mononuclear cells (PBMCs) was measured to assess the toxicity of 20 µg/mL and 100 µg/mL of 1018 and its derivatives.

We previously identified 1018 as a potent immunomodulatory peptide with excellent* in vivo* protective and anti-inflammatory activity [28,29,30]. The identification here of 1018 derivatives enabled us to see if the two properties of anti-biofilm and immunomodulatory activity were conserved in the new 1018 derivatives. Therefore, we assessed the relative ability of HE1, HE4, HE10, and HE12 cf. 1018 to induce monocyte chemotactic protein-1 (MCP-1), a key chemokine that promotes recruitment and infiltration of monocytes and macrophages [17]. At 20 and 100 µg/mL, peptides HE1, HE4, and HE10 significantly induced MCP-1 in human peripheral blood mononuclear cells (PBMCs) to the same (or greater) extent as 1018 *cf*. the untreated control (Figure 4A). In contrast, peptide HE12 was virtually inactive. None of these peptides showed substantial cytotoxicity as assessed by the release of cytosolic lactate dehydrogenase (LDH) (Figure 4C). As persistent lower airway infection contributes to the lung pathology exhibited in cystic fibrosis, the peptides were also assessed for their ability to reduce the production of tumor necrosis factor-alpha (TNF-α) in response to pro-inflammatory bacterial lipopolysaccharides (LPS). Interestingly all HE peptides substantially reduced—by more than 95%—the levels of LPS-induced TNF-α (Figure 4B). These results indicate that peptides HE4 and HE10, like 1018, are not only potent anti-biofilm agents, but also possess notable immunomodulatory potential by selectively enhancing chemokine production while balancing the pro-inflammatory response.

## 3. Discussion

Currently, there are no clinically approved antimicrobial agents that target bacterial biofilms. Previously, we described that the mechanism of action of broad-spectrum anti-biofilm peptide 1018 involved binding and degradation of the second messenger nucleotides (p)ppGpp, which are involved in biofilm development [22]. In this report, we extended these studies on anti-biofilm peptide 1018 to examine whether this peptide inhibited biofilms formed by clinical strains isolated from CF patients. In addition, we examined if the dual properties of immunomodulatory and anti-biofilm activity were conserved in 1018 derivatives, since both of these activities have been reported to be independent of antimicrobial activity* vs.* planktonic cells [17,21,30].

It was demonstrated that 10 µg/mL of 1018 inhibited biofilm formation by most strains screened (Table 1), which was generally well below the MIC* vs.* planktonic cells (Table 1). Indeed, consistent with previous studies [21], all *Burkholderia* genomovars were found to be highly resistant to peptides 1018, LL-37, and 1037, as well as to the cationic antibiotic polymyxin B (Table 1). Against *P. aeruginosa* clinical strains, the peptides showed modest to very weak antimicrobial activity in the order 1018 > LL-37 > 1037.

Biofilm formation by most *P. aeruginosa* isolates (Table 2) was substantially inhibited by 10 µg/mL of 1018, except strain 7632C, for which biofilm formation was only inhibited by 19% (Table 2). Biofilm formation by *P. aeruginosa* mucoid strain 4608 was affected the most by peptide action (67% inhibition). Peptide 1018 also inhibited biofilm formation by at least 52% in all *Burkholderia* CF isolates tested, with *B. multivorans* D2661 being the most susceptible strain (75% inhibition) (Table 2). Additional experiments using a hydroxyapatite disc biofilm model revealed that the peptide (10 µg/mL) triggered cell death in biofilms formed by both *P. aeruginosa* PA14 and *B. cenocepacia* (IIIa) 4813 (Figure 1 and Table 3), although interestingly, 1018-induced death of *B. cenocepacia* biofilms had not been previously observed using the flow cell biofilm assay [22]. However, *B. cenocepacia* cell death, as observed here, might reflect the different model systems. Killing was further confirmed using scanning electron microscopy (Figure 1C). Thus, the biofilm inhibitory activity of peptide 1018 (Table 2) might be due in part to the ability of 1018 to induce biofilm cell death (Figure 1 and Figure 2; Table 3).

Peptide 1018 has two independent activities, as an immunomodulator [28,29,30] and as an antibiofilm agent [22]. To understand if these properties were evident in related peptides we designed a series of 1018 derivatives. The best of these showed similar (HE4) or reduced (HE10) anti-biofilm activity against *P. aeruginosa* (Figure 2) but enhanced activity* vs.* the Gram-positive organism *S. aureus* (Figure 3), and generally conserved the broad-spectrum activity of the parent peptide 1018. Most intriguing was the effect of deletion of the hydrophobic residues valine and alanine in peptide HE10 which decreased anti-*Pseudomonas* activity but increased anti-MRSA activity observed. Peptide HE12, which was the same length as HE10 and had the same balance of charged and hydrophobic amino acids, lacked anti-biofilm activity against both the tested Gram-negative and Gram-positive bacteria (Figure 2 and Figure 3), demonstrating the importance of the amino acid sequence rather than composition. Interestingly, peptides HE4 and HE10 retained the ability of 1018 to modulate the immune response, as they enhanced the production of chemokine MCP-1 (Figure 4A) while strongly suppressing the production of TNF-α (Figure 4B), an anti-inflammatory property. Conversely, peptide HE12 did not significantly stimulate MCP-1 production (Figure 4A), but did inhibit the release of TNF-α from PBMCs (Figure 4B). This strongly suggests that chemokine induction and suppression of pro-inflammatory cytokines is independently determined, as proposed earlier [35]. Importantly, none of the amino acid modifications tested led to increased cytotoxicity of the peptides (Figure 4C).

In conclusion, we report that anti-biofilm peptide 1018 inhibited biofilm formation by multiple clinical isolates from CF patients. Moreover, 1018 derivative HE4 showed equivalent activity* vs.* *Pseudomonas* and enhanced anti-biofilm activity against MRSA while retaining the immunomodulatory properties of parent peptide 1018. On the other hand, peptide HE12 provided a potential negative control since it lacked anti-biofilm activity and did not substantially stimulate MCP-1 production. Since *P. aeruginosa* and *Burkholderia* strains are the most prevalent pathogens in the CF lung and are known to form biofilms, our findings suggest that peptide 1018 and its derivatives may be used in combination with conventional antibiotics [27] against CF-related inflammatory lung infections.

## 4. Experimental

### 4.1. Peptide Synthesis

Peptides used in this study were synthesized by GenScript (Piscataway, NJ, USA) using solid-phase 9-fluorenylmethoxy carbonyl (Fmoc) chemistry and made >95% pure using reverse-phase high-performance liquid chromatography (HPLC). Peptide mass was confirmed by mass spectrometry.

### 4.2. Bacteria, Growth, and Assessment of Minimal Inhibitory Concentrations (MIC)

In addition to the wild type lab isolates *Pseudomonas aeruginosa* strains PAO1 and PA14, a variety of cystic fibrosis clinical isolates of *P. aeruginosa* and *Burkholderia cepacia* complex strains were utilized (Table 1). Planktonic cells from each of the *P. aeruginosa* and* B. cepacia* strains were grown in BM2 glucose or cation-adjusted Mueller-Hinton. The minimal inhibitory concentration (MIC) for peptides in brain-heart infusion (BHI) was assessed by the broth microdilution method with minor modifications for cationic peptides [21]. Peptide 1018 was dissolved in water and stored in glass vials at −20 °C. MIC assays were performed in sterile 96-well polypropylene microtiter plates. Peptide 1018 was added to the plate at increasing concentrations and *P. aeruginosa* or* B. cepacia* bacteria were inoculated to a final concentration of 5 × 10^5^ colony forming units (CFU)/mL per well. The plates were incubated at 37 °C for 18 h. The MIC was defined as the lowest concentration of peptide at which no planktonic growth was observed.

### 4.3. Biofilm 96-Well Plate Assays

Biofilm formation was initially analyzed using a static abiotic solid surface assay as described elsewhere [21]. Dilutions (1/100) of overnight cultures were incubated in BM2 glucose medium (62 mM potassium phosphate buffer (pH 7), 7 mM (NH_4_)_2_SO_4_, 2 mM MgSO_4_, 10 μM FeSO_4_, 0.4% (wt/vol) glucose, 0.5% (wt/vol) Casamino Acids) in polypropylene microtiter plates (Fisher Scientific, Waltham, MA, USA) in the presence of the different peptides for 22 h at 37 °C. Planktonic cells were removed and biofilm cells adhering to the side of the tubes were stained with crystal violet, extracted into ethanol, and the optical density at 595 nm (OD_595_) measured using a microtiter plate reader (Bio-Tek Instruments Inc., Winooski, VT, USA). Peptides were added at time zero (prior to adding the diluted, overnight cultures) at various concentrations, and the decrease in biofilm formation was recorded at 22 h for *P. aeruginosa*, *Burkholderia* and Methicillin-resistant *Staphylococcus aureus* isolates. Peptides 1018, HE4, HE10 and HE12 at 10 µg/mL, and peptides HE1, HE2 and HE3 at 15 µg/mL (due to their lesser activity), were investigated using the BioFlux apparatus [36].

### 4.4. Culturing Biofilms Using the Hydroxyapatite Biofilm Model 

Sterile hydroxyapatite (HA) disks (0.38-inch diameter by 0.06-inch thickness; Clarkson Chromatography Products, Williamsport, PA, USA) were used as a biofilm substrate.* P. aeruginosa* or* B. cepacia* strains isolated from cystic fibrosis (CF) patients were used as the test organisms and grown at 37 °C overnight on BHI agar (Becton-Dickinson, Sparks, MD, USA) plates and then suspended in BHI broth at a spectrophotometrically-standardized OD_405_ = 0.10 (3.0 × 10^7^ CFU/mL). The HA disks were placed in the wells of a 24-well tissue culture plate containing 1.8 mL of BHI. Each well was inoculated with 0.2 mL of the above *P. aeruginosa* or* B. cepacia* suspensions. The discs were then incubated at 37 °C for 3 days to allow biofilm growth prior to peptide treatment. The HA disk is a well-established platform for biofilm growth [37]. Previous studies have shown that HA does not affect the viability of bacteria within biofilms [38] and indeed most of these bacteria remain viable for months [38]. Further, HA is the component of human bone tissue, which makes it more biocompatible and less cytotoxic compared to other surfaces such as glass. Thus, HA is less likely to affect the susceptibility to peptides.

### 4.5. Anti-Biofilm Effect of Peptide 1018 Evaluated Using the Hydroxyapatite Biofilm Model

After the formation of a 3-day-old biofilm, the culture medium of each well was replaced by 1.98 mL of fresh BHI. The biofilm-covered HA disks were subjected to peptide 1018 treatment by adding 20 μL of 1.0 mg/mL peptide (or 20 μL of water as a negative control) into each well (1.98 mL BHI) to achieve a final concentration of 10 μg/mL. HA disks were treated for 72 h at 37 °C (the same concentration of peptide solution was replenished every 24 h).

### 4.6. Confocal Laser Scanning Microscopy

*P. aeruginosa* or* B. cepacia* biofilms on HA disks, including peptide exposed and control disks, were rinsed in 0.85% physiological saline for 1 min and subjected to bacterial viability staining and confocal laser scanning microscopy as previously described [22,34].

### 4.7. Scanning Electron Microscopy Examination of Hydroxyapatite-Grown Biofilms

Three additional *P. aeruginosa* and *B. cepacia* biofilms treated with 10 µg/mL peptide 1018 for 72 h, or the equivalent amount of water as a negative control, were collected for scanning electron microscopy examination. Samples were prefixed with phosphate-buffered 2.5% glutaraldehyde for 10 min before further fixation in 1% osmium tetroxide for 1 h. The specimens were then subjected to increasing concentrations of ethanol (50%, 70%, 80%, and 100%) for dehydration. The dehydrated specimens were dried by using a critical point drier (Samdri-795; Tousimis Research Corporation, Rockville, MD, USA), sputter-coated with gold palladium (Hummer VI; Technic Inc, Anaheim, CA, USA), and examined by SEM (Helios Nanolab 650, FEI, Eindhoven, The Netherlands) at an accelerating voltage of 5 kV using 20,000× magnification.

### 4.8. Biofilm Cultivation in Flow Cell Chambers and Microscopy

Biofilms were grown for 72 h, in the absence or presence of the desired concentration of the different peptides, at 37 °C in flow chambers with channel dimensions of 1 by 4 by 40 mm, and then stained and examined by confocal microscopy, as described previously [22]. For biofilm prevention studies the peptide was present in the flow-through medium throughout the biofilm growth while for eradication experiments, biofilms were allowed to grow for 2 days, before treating them with peptide for the last 24 h of the experiment.

### 4.9. Immune Modulation Studies

These studies were done as described previously [28]. Briefly, human peripheral blood mononuclear cells (PBMCs) were isolated from the blood of healthy individuals obtained under University of British Columbia (UBC) human ethics approval. PBMCs were diluted to 2 × 10^6^ cells/ml in RPMI-1640 medium (Gibco^®^, Grand Island, NY, USA) supplemented with 10% fetal bovine serum (FBS). Subsequently, 250 µL of cells were seeded in each well of a 48-well dish. PBMCs were allowed to incubate for one hour at 37 °C in 5% CO_2_, and then ether-washed peptides HE4, HE10, HE12 or 1018 were subsequently added to give a final peptide concentration of 20 μg/mL or 100 μg/mL. To assess chemokine induction after 24 h, the levels of chemokine MCP-1 (CCL2) were assessed in the supernatants three times independently using a sandwich ELISA kit (BioSource International, Grand Island, NY, USA). To look at the anti-inflammatory activity of the peptides in suppressing LPS-induced TNFα, *P. aeruginosa* LPS was added at a final concentration of 20 ng/mL as well as peptides at 20 μg/mL or 100 μg/mL. Secretion of TNF-α was monitored by capture ELISA (eBiosciences, San Diego, CA, USA) after 24 h.

### 4.10. Cytotoxicity Assessment 

To assess peptide-mediated cytotoxicity, human PBMCs (1 × 10^5^) were seeded into 96-well plates (Sarstedt, Newton, NC, USA) in RPMI-1640 medium and incubated for one hour at 37 °C in 5% CO_2_ before peptide treatment. The release of the cytosolic enzyme lactate dehydrogenase (LDH) was then measured after 24 h. Untreated control cells were used as a reference for normalization. All experiments were performed in triplicate.

## 5. Conclusions

Our study extends our research on anti-biofilm peptide 1018. Here we show that this peptide prevents biofilm formation, eradicates mature biofilms and kills biofilms formed by a wide range of *P. aeruginosa* and *B. cenocepacia* strains isolated from cystic fibrosis patients. New peptides derived from 1018 showed similar or decreased anti-biofilm activity against *P. aeruginosa* biofilms, but increased activity against biofilms formed by the Gram-positive bacterium methicillin resistant *Staphylococcus aureus*. In addition, some of these new peptide derivatives retained the immunomodulatory activity of 1018 and were non-toxic towards human PBMCs. The combined anti-biofilm and immunomodulatory activities of the peptides described here make them promising therapeutic candidates for the treatment of antibiotic-resistant infections.
